# Case of Severe Injury of the Head

**Published:** 1830-02

**Authors:** J. C. Cox

**Affiliations:** Surgeon


### INJURY OF THE HEAD.

### *Case of severe Injury of the Head.* By J. C. Cox, Surgeon, F.L.S.

It is well known that in cases of injury of the head, there is no symptom of more fearful augury than bleeding from the ear; so much so, that there are few exceptions to its being the indication of a fatal termination. The following case, which was attended with very serious symptoms, is a happy exception to this generally received opinion.

A gentleman, after dining a few miles out of town, where he drank pretty freely, was, on his return home, thrown out of his stanhope, and fell on his head. He was taken up insensible, was placed in a hackney coach, and conveyed to his house in town. I was called to him at about one o'clock in the morning, and found him lying on the sofa, in a state of stupor. He was, however, easily roused, and answered questions rationally. After removing him to his chamber, I proceeded to examine into the nature of the injury he had received. I found a severe contusion on the back part of the head; the scalp exceedingly swoln, from extravasated blood, and a small superficial wound. There was considerable bleeding from the left ear, and the face was paralysed on the left side: there was, however, no loss of sensation, so that the mischief was in the portio dura. The pupils were dilated. There was no other mark of injury excepting on the head.

I dilated the wound of the scalp, thinking it probable there might be fracture of the skull. This, however, was not discovered; but the incision removed the extreme swelling of the scalp, and gave great relief. As the pulse was eighty, full, and strong, I bled the patient to twenty ounces, and then put him to bed. I ordered some pills of calomel and jalap immediately, and a dose of salts and senna every four hours, till the bowels were freely opened.

Sept. 27th.—I found him much better this morning. He was quite tranquil and rational, felt no pain in the head; the medicine had acted well: and he only complained of giddiness on raising his head from the pillow. He complained of air being forced through the ear on using his handkerchief, which made it rather painful. I desired him to take nothing but tea, (to which, I believe, he adhered for some days,) and to continue the aperient medicine. The speech was considerably affected from the paralytic affection of the muscles of the face, and he had no power of raising the eyebrow; the sensation, however, was not at all impaired. The pulse was tranquil, and there were no other bad symptoms.

October 2d.—My patient has gone on favorably for several days past, although I have had some difficulty in making him adhere to a system of abstinence. The face continues in the same distorted state; and I ordered cupping behind the ear to twelve ounces, and a blister.

It is unnecessary to detail the daily progress of the case: I will only state that, after the first ten days, my patient did every thing to make it go on badly, by taking wine to the extent of one, and sometimes two, bottles a day, which happily failed of doing material mischief. The wound of the scalp healed in a few days. It was necessary to order cupping to the nape of the neck, as the pulse became full and strong, from indiscretion in diet. I also ordered leeches to the root of the mastoid process, followed by a blister. This treatment produced considerable improvement in the face, which was gradually recovering its natural appearance when my patient left town for Paris, about a month from the time of the accident.

It is difficult to form an accurate opinion of the exact nature of the injury which produced such alarming symptoms. We can hardly suppose that any serious fracture of the basis of the skull could occur without being fatal; and yet it is difficult to conceive how the portio dura could be so deranged, and the membrana tympani ruptured, without some serious internal mischief. Certainly the latter may be occasioned by severe concussion, but the paralysis of the portio dura is not so easily explained. It may, however, be consolatory to be able to refer to a case of this nature which admits a gleam of hope under the occurrence of symptoms most alarming and discouraging.

33, *Montagu square; December* 1829.

